# Clinically-identified C-terminal mutations in fibulin-3 are prone to misfolding and destabilization

**DOI:** 10.1038/s41598-020-79570-x

**Published:** 2021-02-04

**Authors:** DaNae R. Woodard, Emi Nakahara, John D. Hulleman

**Affiliations:** 1 Department of Ophthalmology, University of Texas Southwestern Medical Center, 5323 Harry Hines Blvd, Dallas, TX USA; 2 Department of Pharmacology, University of Texas Southwestern Medical Center, 5323 Harry Hines Blvd, Dallas, TX USA

**Keywords:** Glycobiology, Mechanisms of disease, Protein folding, Protein translocation, Endoplasmic reticulum, Protein aggregation

## Abstract

Distinct mutations in the secreted extracellular matrix protein, fibulin-3 (F3), have been associated with a number of ocular diseases ranging from primary open angle glaucoma to cuticular age-related macular degeneration to a rare macular dystrophy, Malattia Leventinese (ML). The R345W F3 mutation that causes ML leads to F3 misfolding, inefficient secretion and accumulation at higher intracellular steady state levels in cultured cells. Herein, we determined whether fifteen other clinically-identified F3 mutations also led to similar levels of misfolding and secretion defects, which might provide insight into their potential pathogenicity. Surprisingly, we found that only a single F3 variant, L451F, presented with a significant secretion defect (69.5 ± 2.4% of wild-type (WT) F3 levels) and a corresponding increase in intracellular levels (226.8 ± 25.4% of WT F3 levels). Upon follow-up studies, when this conserved residue (L451) was mutated to a charged (Asp or Arg) or bulky (Pro, Trp, Tyr) residue, F3 secretion was also compromised, indicating the importance of small side chains (Leu, Ala, or Gly) at this residue. To uncover potential inherent F3 instability not easily observed under typical culture conditions, we genetically eliminated the sole stabilizing N-linked glycosylation site (N249) from select clinically-identified F3 mutants. This removal exacerbated R345W and L451F secretion defects (19.8 ± 3.0% and 12.4 ± 1.2% of WT F3 levels, respectively), but also revealed a previously undiscovered secretion defect in another C-terminal variant, Y397H (42.0 ± 10.1% of WT F3 levels). Yet, glycan removal did not change the relative secretion of the N-terminal mutants tested (D49A, R140W, I220F). These results highlight the uniqueness and molecular similarities between the R345W and L451F variants and also suggest that previously identified disease-associated mutations (e.g., R140W) are indistinguishable from WT with respect to secretion, hinting that they may lead to disease by an alternative mechanism.

## Introduction

Vision loss significantly affects the quality of life of over 200 million middle-aged and elderly people worldwide^1^. In the United States alone, it is estimated that by the year 2050, the number of people who will suffer from either uncorrectable vision defects or blindness will double to more than 20 million^2^. Thus, there is a need for effective ocular therapies for the aging population and a deeper understanding of the environmental and genetic factors that influence disease^3–5^. Age-related macular degeneration (AMD) is the leading cause of progressive and irreversible vision loss in individuals over the age of 65 in industrialized nations^6^. AMD is a late-onset disease that results in the degeneration of photoreceptor and retinal pigment epithelial (RPE) cells, compromising an individual’s ability to retain sharp central vision^7,8^. The most common clinical hallmark of early-stage dry AMD is the formation of yellow, extracellular lipid and protein deposits underneath the RPE known as drusen^8–10^. The accumulation of drusen eventually correlates with the atrophy of RPE cells followed by the dysfunction of photoreceptor cells, causing irreversible blindness^11,12^. Dry AMD makes up about 85–90% of AMD cases and there are currently no effective therapies^13,14^.

Although AMD is an etiologically complex disease due to a variety of genetic and environmental risk factors^15–17^, insight into its pathogenesis can be gained by studying phenotypically similar, early-onset monogenic macular diseases. One such disease is Malattia Leventinese (ML), a rare macular dystrophy caused by an autosomal dominant Arg345Trp (R345W) mutation in the fibulin-3 (F3) protein, wherein patients develop AMD-like symptoms including drusen formation as early as 20 years of age^18,19^. Increasing evidence suggests that F3 is involved in AMD including: (1) the accumulation of wild-type (WT) F3 surrounding drusen in AMD patient donor eyes^20^, but not around drusen of asymptomatic patients, (2) increased copy number variants near the *EFEMP1* promoter (the gene that encodes for F3) were linked to increased risk for AMD^21^, and (3) a sequence variant, Asp49Ala (D49A), was discovered in a patient with cuticular drusen, a clinical subtype of AMD^22^. The combination of these findings strongly suggests that both mutant and WT F3 may play an important role in the development of AMD or AMD-like retinal dystrophies^23^.

F3 is a 55 kDa secreted extracellular glycoprotein that belongs to the fibulin family of proteins^24^. While broadly expressed throughout the body during development, F3 is highly expressed in various ocular tissues including the retina and RPE^24–26^. Several in vitro studies have shown that the R345W F3 mutant, which causes ML, is misfolded, results in a secretion defect, and disrupts protein homeostasis^20,27–30^. In vivo studies have shown that R345W F3 knock-in mice develop basal laminar deposits underneath the RPE, akin to drusen observed in humans^31,32^. Although these in vitro and in vivo studies examined and characterized the effect of the R345W variant, additional F3 variants have been identified in the human population, some of which have been linked to disease. These mutations, including the D49A variant (mentioned above), the R140W variant found in a family with primary open-angle glaucoma^33^, and the C55R variant found in two patients with recessive Marfanoid syndrome^34^, have essentially remained uncharacterized. Additional mutations have been identified in the *EFEMP1* gene in patients with ocular disorders (information obtained through ClinVar^35^), but the clinical significance of these genetic alterations is unclear.

We postulated that secretion defects, as seen with the R345W mutation, may be a universal mechanism by which F3 variants ultimately contribute to ocular disease. In the work we describe herein, we selected fifteen additional clinically-idenitified F3 variants (https://www.ncbi.nlm.nih.gov/clinvar), and evaluated their secretion and intracellular accumulation propensities in HEK293A and ARPE-19 cells. New variants that were identified with secretion defects were next evaluated for their ability to activate the unfolded protein response (UPR) and the molecular basis responsible for retention was identified at the amino acid level. Lastly, we determined whether N-linked glycosylation of F3 serves as a unifying stabilizing force, enabling the efficient secretion of subtly unstable mutants.

## Results

**Figure 1:**
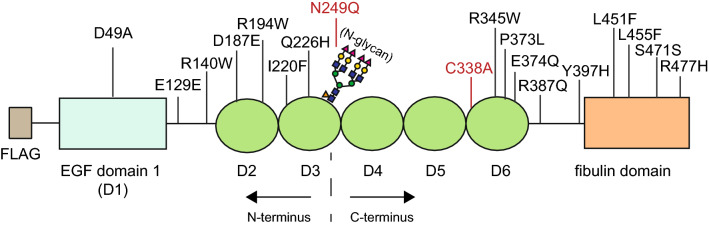
Schematic of clinically-identified F3 variants with respect to localization of F3 domains.

Previously, we and others have demonstrated that the R345W F3 variant, which causes ML, is inefficiently secreted from cultured cells^20,27^. We rationalized that additional F3 mutations may also cause secretion defects, serving as a potential universal mechanism by which distinct F3 variants contribute to ocular disease. Using ClinVar, a database that lists mutations present in patients within the human population, we selected fifteen additional clinically-identified, uncharacterized/poorly characterized variants identified in F3 (Fig. 1). The *EFEMP1* gene that encodes for F3 is one of many genes included within retinal dystrophy panels (https://www.ncbi.nlm.nih.gov/gtr/tests/522537/, https://www.egl-eurofins.com/tests/?testid=MM239) which are typically prescribed for patients with unknown ocular diseases. We selected a range of F3 missense mutations which have been previously reported in either patients with ocular diseases (D49A, R140W, Y397H, L451F) or identified through other means (remainder). We next recorded their clinical significance (ClinVar), determined their potential pathogenicity (PROVEAN and PolyPhen), and allele frequency (gnomAD, Table 1). As expected, the R345W variant was classified as pathogenic in ClinVar and in silico prediction indicated that it is deleterious (PROVEAN score of − 3.309) and probably damaging (PolyPhen score of 1.000, Table 1). Due to the extent of study of this mutation, along with documented human and mouse model data, we used the R345W mutation as a benchmark for relevance to ocular disease. However, no other variant was identified as completely fulfilling these three criteria (i.e., simultaneously determined to be pathogenic, deleterious, and damaging using the aforementioned programs), suggesting that additional metrics, such as secretion propensity, may be needed to provide more information regarding pathogenicity. The variant that resembled R345W the closest in the in silico modeling was the Y397H mutation, which was identified as potentially pathogenic (ClinVar), deleterious (PROVEAN score of − 3.276) and probably damaging (PolyPhen score of 1.000, Table 1). The clinical significance of the remaining mutations were determined to be benign, likely benign, conflicting interpretations, or uncertain significance (ClinVar, Table 1). A few variants not listed in ClinVar were I220F (previously identified in a control individual^18^) and N249Q and C338A (engineered mutations not present within the human population). Interestingly, 6 out of 10 mutations classified as variants of unknown significance were predicted to be deleterious (PolyPhen) and/or damaging (PROVEAN) to the structure of F3 (Table 1). In contrast, the synonymous F3 variants were predicted to be non-pathogenic (PROVEAN). Many of the variants we analyzed were found at surprisingly high frequencies, including the synonymous E129E variant (most common coding variant, 2.73 × 10^−2^), the R387Q variant (most common missense variant, 1.14 × 10^−3^), followed closely by the D49A variant (1.05 × 10^−3^, Table 1).

**Table 1:** Clinical signficance, predicted stability scores, and allele frequencies of F3 variants.

F3 variant	ClinVar: clinical significance	PROVEAN: impact prediction	PolyPhen 2.0: functional effect prediction	gnomAD: allele frequency
D49A	Conflicting interpretations of pathogenicity	0.384; neutral	0.002; benign	1.05 × 10^−3^
E129E	Benign	0.000; neutral	N.A	2.73 × 10^−2^
R140W	Uncertain significance	− 1.580; neutral	0.997; probably damaging	7.95 × 10^−6^
D187E	Uncertain significance	− 0.728 neutral	0.389; benign	3.98 × 10^−6^
R194W	Uncertain significance	− 3.802; deleterious	1.000; probably damaging	1.99 × 10^−5^
I220F	N.D	0.904; neutral	0.000; benign	5.97 × 10^−5^
Q226H	Uncertain significance	2.873; neutral	0.000; benign	N.D
N249Q*	N.A	− 3.965; deleterious	1.000; probably damaging	N.A
C338A*	N.A	− 8.812; deleterious	0.999; probably damaging	N.A
R345W	Pathogenic	− 3.309; deleterious	1.000; probably damaging	N.D
P373L	Uncertain significance	− 1.378; neutral	0.004; benign	5.67 × 10^−5^
E374Q	Uncertain significance	− 2.287; neutral	0.995; probably damaging	3.98 × 10^−6^
R387Q	Likely benign	− 2.072; neutral	0.219; benign	1.14 × 10^−3^
Y397H	Likely pathogenic	− 3.276; deleterious	1.000; probably damaging	N.D
L451F	Uncertain significance	− 3.577; deleterious	0.996; probably damaging	1.14 × 10^−5^
L455F	Uncertain significance	− 2.539; deleterious	1.000; probably damaging	6.36 × 10^−5^
S471S	Uncertain significance	0.000; neutral	N.A	1.98 × 10^−4^
R477H	Uncertain significance	− 3.619; deleterious	1.000; probably damaging	1.41 × 10^−5^

*Indicates rationally-designed variant.

### Few F3 mutations cause secretion defects

**Figure 2:**
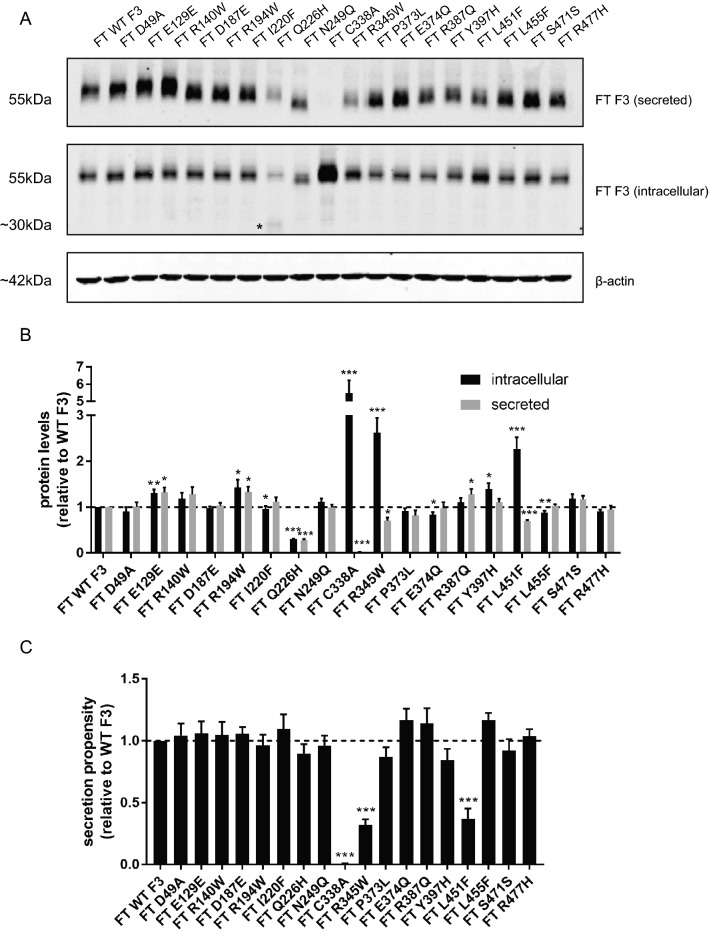
Secretion of F3 variants. (**A**) Western blot of secreted and intracellular levels of F3 variants in HEK293A cells. Asterisk indicates truncated intracellular Q226H band. (**B**) Quantification of secreted, intracellular, and (**C**) secretion propensities of F3 variants shown in (**A**), n ≥ 6, mean ± SEM (*p < 0.05, **p < 0.01, ***p < 0.001, one sample *t* test vs. a hypothetical value of 1 [i.e., unchanged]).

We next determined the secretion propensities of each of the select F3 variants in a cell culture system. Using site-directed mutagenesis, we generated the fifteen new F3 mutations (each construct containing an N-terminal FLAG epitope as described previously^36^, Fig. 1). We then quantified intracellular (cell lysate) and extracellular (secreted) F3 levels from transfected HEK293A cells using western blotting (Fig. 2A,B). An additional metric, secretion propensity, which takes into account both intracellular and secreted F3 (the ratio of WT-normalized secreted protein/intracellular), was also calculated (Fig. 2C). As we have observed previously^27^, C338A F3, a genetically engineered F3 that is not folded due to absence of a required disulfide bond in the 6th calcium-binding EGF domain, and R345W F3 both displayed significant defects in secretion propensity (0.007 ± 0.003 [*p* < 0.001], 0.32 ± 0.04 [*p* < 0.001] of WT F3 levels respectively, Fig. 2A,C). Interestingly, out of the fifteen newly tested variants, only L451F displayed a significant reduction in secretion propensity (0.38 ± 0.08 [*p* < 0.05] of WT F3 levels), suggesting that this variant may be misfolded (Fig. 2A,C). These secretion defects were not due to reduced transfection efficiency or expression levels (Sup. Figure 2A).

Surprisingly, we observed that the Q226H variant resulted in significantly reduced intracellular (30.2 ± 0.9% [*p* < 0.001] of WT) and secreted levels (27.4 ± 2.9% [*p* < 0.001)] of WT), which were accompanied by a significant concomitant reduction in *EFEMP1* transcript levels (23.3 ± 0.9% of WT, Sup. Figure 2A). Even more surprising was that in addition to forming full length F3, this variant formed an intracellular truncated product of ~ 30 kDa through an unknown mechanism (asterisk, Fig. 2A). While Q226H has intriguing characteristics, it did not fall into our criteria for identifiying mutants that behave similarly to R345W, thus we chose not to focus on this variant in this paper.

### Secretion propensities of F3 in ARPE-19 cells

**Figure 3:**
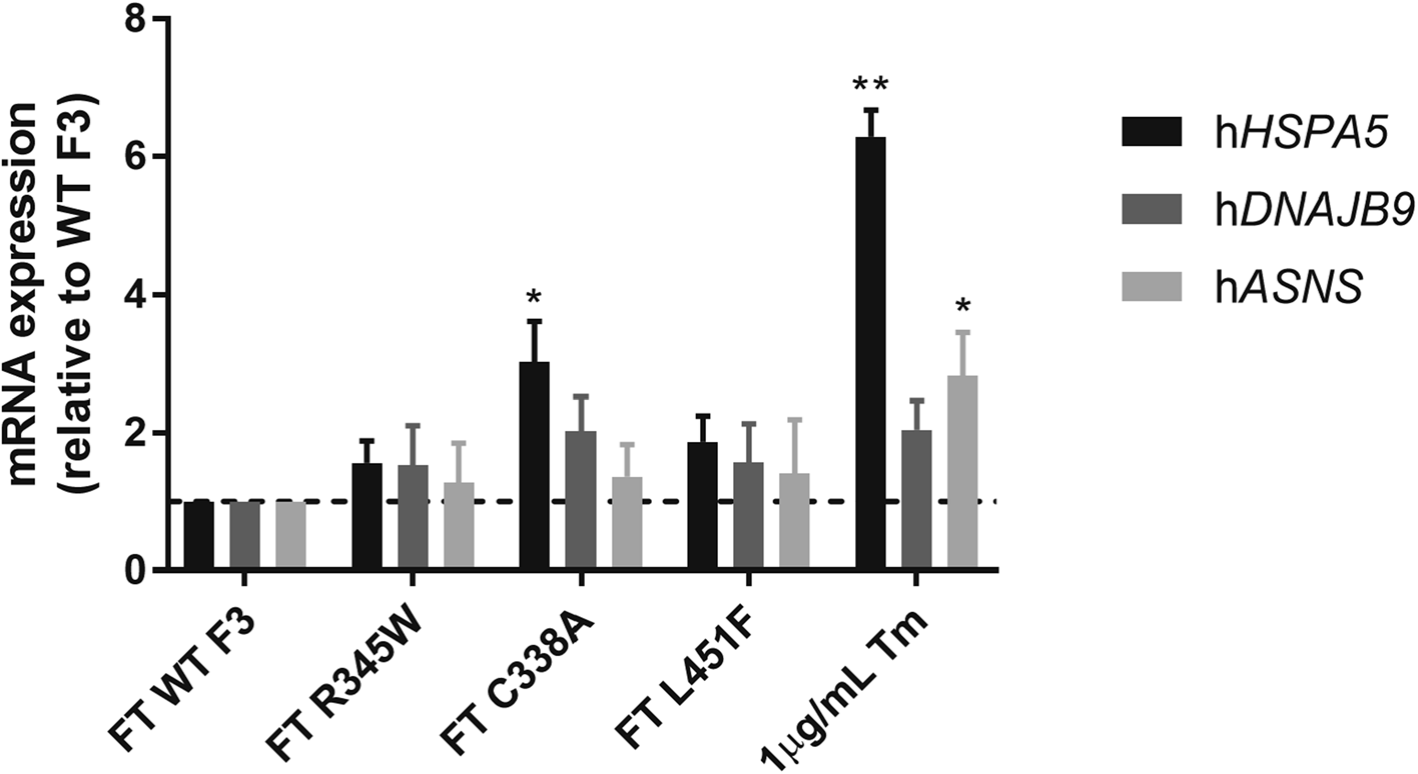
L451F does not elicit an ER stress response. qPCR analysis of h*HSPA5*, h*DNAJB9*, and h*ASNS* transcript levels with TaqMan probes in WT F3, R345W, C338A, L451F–expressing HEK293A cells and cells treated with 1 μg/mL tunicamycin (Tm), 24 h. n = 3, mean ± SEM (*p < 0.05, **p < 0.01, one sample *t* test vs. a hypothetical value of 1 [i.e., unchanged]).

Because F3 is expressed in retinal pigment epithelial cells (RPE) in the eye^24,25^, we next determined whether F3 secretion propensity defects were also apparent in an RPE-based cell culture system using ARPE-19 cells (Sup. Figure 3A-C). We selected disease-associated variants as well as those with secretion defects and stably expressed them in ARPE-19 cells. Upon expression of WT, D49A, R140W, R345W, Y397H, and L451F F3 in ARPE-19 cells (Fig. 3), we detected significantly lower secretion propensities for R345W (0.21 ± 0.04 [*p* < 0.001]) and L451F (0.44 ± 0.08 [*p* < 0.001]) (Sup. Figure 3C), a phenomenon similar to what was observed in HEK293A cells (Fig. 2A–C). Furthermore, analysis of these stable cells by qPCR demonstrated no significant changes in *EFEMP1* transcript levels (Sup. Figure 2B).

### L451F does not induce an endoplasmic reticulum (ER) stress response

Next, we determined whether an ER stress response was induced due to significantly higher intracellular levels of C338A, R345W, or L451F F3. HEK293A cells were transfected with WT, C338A, R345W, or L451F F3 constructs or treated with tunicamycin (Tm) as an unfolded protein response (UPR) positive control. We performed qPCR using select TaqMan probes (*HSPA5*, *DNAJB9*, and *ASNS*), the levels of which are representative of the triggering of select arms (i.e., ATF6, IRE1 and PERK, respectively) of the UPR. We found that only cells expressing the severely misfolded C338A F3 variant demonstrated a significant increase in *HSPA5* transcript levels compared to cells expressing WT F3 (302.2 ± 34.2%, Fig. 3), suggesting that this variant induces ER stress. We observed no significant induction of ER stress in cells expressing R345W or L451F F3 (Fig. 3), suggesting that intracellular levels of these variants are not sufficient to disrupt ER homeostasis. These findings are consistent with our previous results^36^, but in contrast to other findings^28^. These discrepancies are likely due to the method of DNA introduction, expression level differences, and/or different culture systems.

### Charged and aromatic substitutions at position 451 affect F3 secretion

**Figure 4:**
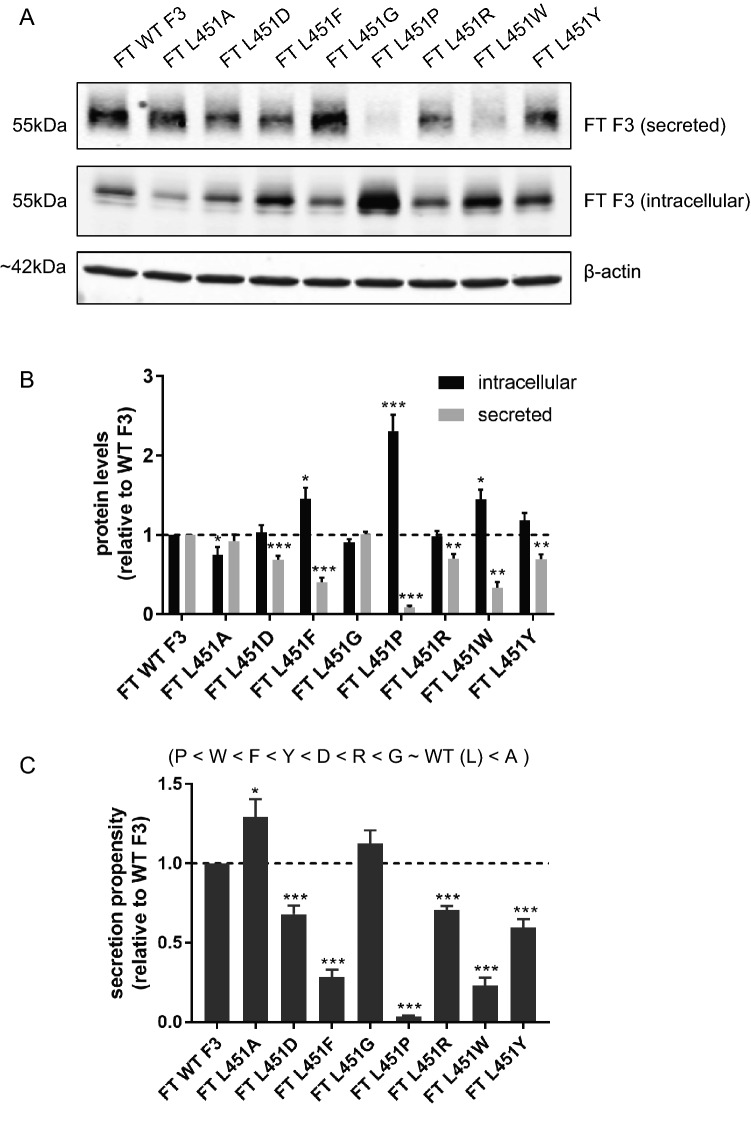
Substitution of leucine at position 451 with select residues. (**A**) Western blot of secreted and intracellular levels of L451F substitution variants. (**B**) Quantification of secreted, intracellular, and (**C**) secretion propensities of L451F substitution variants in (**A**), n ≥ 4, mean ± SEM (*p < 0.05, **p < 0.01, ***p < 0.001, one sample *t* test vs. a hypothetical value of 1 [i.e., unchanged]).

Previously, we demonstrated that substitution of F3 at R345 with aromatic residues or proline also resulted in secretion defects^27^, indicating that the common Arg-to-Trp mutation was not unique in its ability to reduce secretion, but that presence of other sterically-hindering or bulky amino acids would also cause secretion defects. We next decided to explore the molecular basis of how the L451F mutation disrupts F3 secretion in a similar manner. To accomplish this, we generated a panel of substitutions at L451 with diverse amino acid backbones, transfected them into HEK293A cells, and performed western blotting (Fig. 4A). Mutation from the native leucine residue to other small uncharged residues such as alanine (L451A) or glycine (L451G) did not affect secretion compared to WT F3 (92.0 ± 8.4% and 100.6 ± 3.6% of WT levels, respectively) (Fig. 4A,B). Replacement of L451 with charged residues such as aspartic acid (L451D) or arginine (L451R) significantly lowered secretion (68.6 ± 5.0% [*p* < 0.001], 70.0 ± 6.0% [*p* < 0.01] of WT levels, respectively) (Fig. 4A,B). Interestingly, mutation of L451 to a tryptophan (L451W) or tyrosine (L451Y) both resulted in secretion defects (33.3 ± 7.7% [*p* < 0.01], 61.3 ± 2.3% [*p* < 0.01] of WT F3 levels, respectively) (Fig. 4A,B). Substitution of leucine to proline (L451P) resulted in an even more drastic secretion defect (10.3 ± 4.4% [*p* < 0.001] of WT F3 levels, respectively) (Fig. 4A,B), possibly due to proline’s conformationally-restrictive backbone^37,38^. The rank-order of secretion propensity for these L451 variants was Pro < Trp < Phe < Tyr < Asp < Arg < Gly ~ Leu (WT) < Ala (Fig. 4C). Together, these data suggest that, similar to substitutions made at the 345 position, bulky aromatic and restrictive residues at the 451 position are sufficient to cause secretion defects in F3. Supporting this notion, L451 is well-conserved in F3 proteins across species, and reasonably conserved among other human fibulins (Sup. Figure 4A, B). Whereas aromatic residues at the 345 position appeared to disrupt disulfide bond formation within the 6th EGF domain^27^, possibly causing misfolding of that particular domain, it is unclear why similar residues would also disrupt the folding of the fibulin-type domain where the L451 residue is located. Additional studies will have to be performed to begin to tease out such information.

### The N249 N-linked glycan is required for efficient L451F secretion and stabilizes the Y397H variant

**Figure 5:**
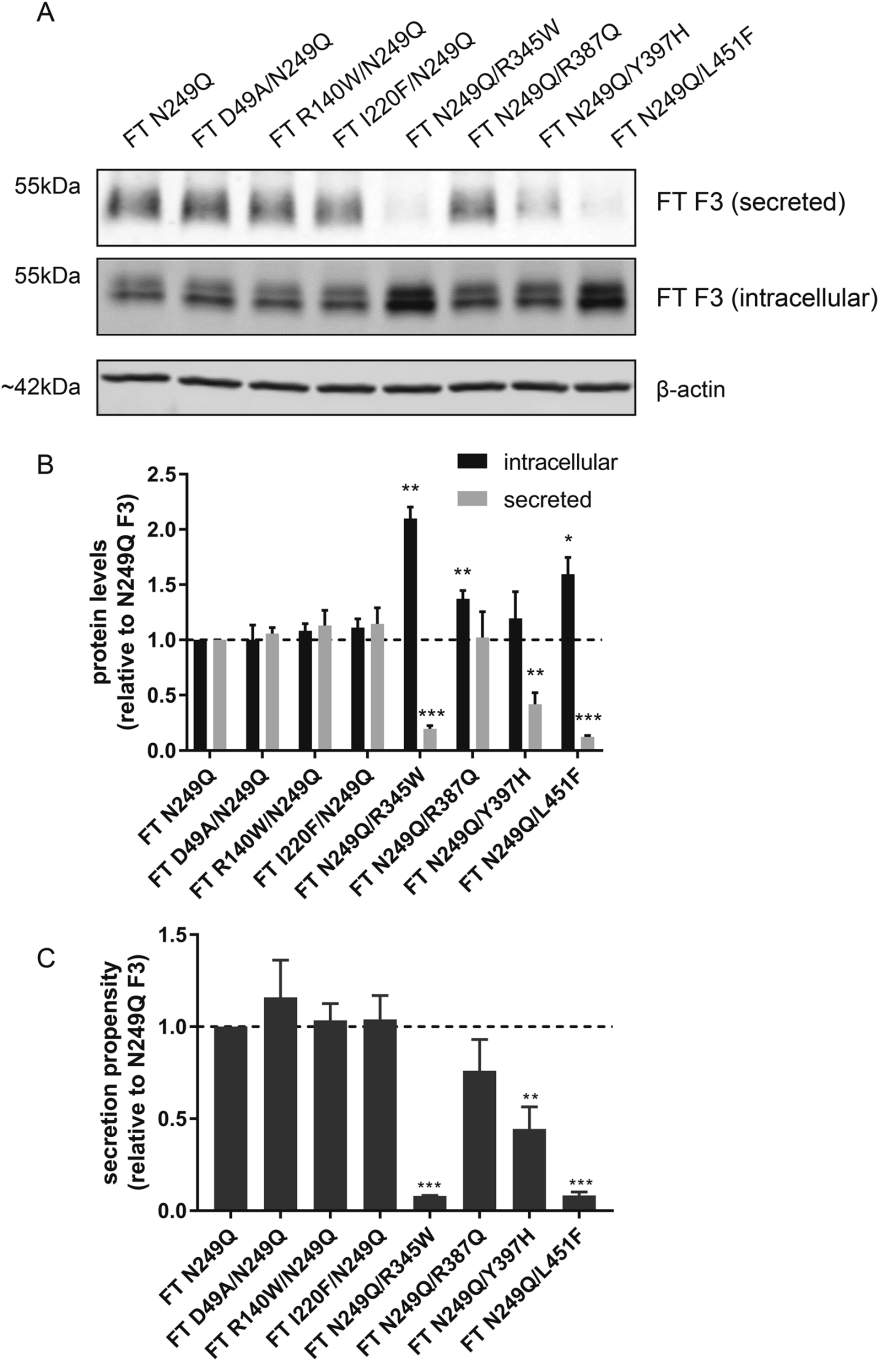
Secretion of F3 variants upon genetic ablation of the N-linked glycan. (**A**) Western blot of secreted and intracellular levels of N249Q F3 variants. (**B**) Quantification of secreted, intracellular, and (**C**) secretion propensities of N249Q F3 variants in (**A**), n ≥ 4, mean ± SEM (*p < 0.05, **p < 0.01, ***p < 0.001 one sample *t* test vs. a hypothetical value of 1 [i.e., unchanged]).

Absence of N-linked glycosylation of R345W F3 leads to increased intracellular aggregation and an altered conformation of R345W F3, suggesting that the N-linked glycan is required in order for R345W to maintain a stable, native-like structure^36^. Due to the molecular similarities between R345W and L451F (i.e., mutation to aromatic residues, degree of secretion deficiency, poor tolerance of other amino acids at the position), we postulated that N-linked glycosylation is also a stabilizing force for L451F as well as additional F3 mutants. To cleanly eliminate the F3 N-linked glycosylation site, we mutated Asn249 to Glu (N249Q) in WT F3 (Fig. 2A) as well as in a series of variants analyzed in Fig. 5. Upon elimination of the glycan itself in WT F3 (designated as N249Q), we did not observe a significant change in secretion (98.8 ± 6.8%) or intracellular (111.8 ± 7.0%) levels of N249Q compared to WT F3 (Fig. 2A,B). However, we did observe a significant reduction in N249Q/R345W and N249Q/L451F secretion (15.8 ± 4.6 [*p* < 0.001] and 12.4 ± 1.2% [*p* < 0.001], respectively, of N249Q F3 levels) (Fig. 5A–C), especially when compared to fully glycosylated R345W and L451F (compare Fig. 5A–C to Fig. 2A–C). Surprisingly, in the absence of N-linked glycosylation, N249Q/Y397H displayed a significant secretion defect (42.0 ± 10.1% [*p* < 0.05] of N249Q F3 levels) (Fig. 5A–C) that was not previously observable in the glycosylated Y397H variant (Fig. 2A–C). This observation suggests that the Y397H variant may exhibit a degree of instability which is compensated by the presence of the N-linked glycan at N249. This compensation may be in the form of intrinsic stabilization due to the glycan itself and/or extrinsic stabilization afforded by promoting interactions of F3 with glycan-binding lectins (e.g., calreticulin and calnexin). The secretion of the remaining variants, D49A/N249Q, R140W/N249Q, I220F/N249Q, and N249Q/R387Q were identical to N249Q F3 (Fig. 5A–C).

## Discussion

In this study, we selected a panel of poorly-characterized, clinically-identified synonymous and missense F3 mutations present in the human population and determined whether these mutations disrupted F3 folding and secretion. Upon initial evaluation, we found that only R345W and L451F F3 displayed a significant secretion propensity defect compared to WT F3. Subsequent follow-up studies demonstrated a broad intolerance for charged and bulky amino acid side chains at the L451 position, and demonstrated a reliance of L451F and another C-terminal variant, Y397H, on a stabilizing N-linked glycan at N249. Interestingly, many of the N-terminal variants we tested that had no effect on F3 secretion were also of a similar molecular nature to the compromised C-terminal variants (i.e., R140W and R194W versus R345W, and I220F versus L451F), hinting that the positioning of the bulky aromatic variations is important and can compromise secretion in a context-dependent manner, an idea which is supported by our previous studies^39^. It is important to note that since no well-established functional assay exists for the evaluation of F3 variants, we believe that monitoring the secretion and intracellular levels of F3 in cell culture provides a reasonable metric for assessing its degree of folding and its potential to trigger cellular dysfunction and/or influence disease.

Our findings also suggest that the D49A and R140W variants behave differently than R345W or L451F. Given the prevalence of the D49A variant in the human population, its presence in a “predisposition screen in an ostensibly healthy population” (ClinVar), combined with neutral or benign in silico predictions, and our secretion results, we believe that this is likely a non-pathogenic polymorphic variant in F3 which may not be involved in AMD. However, it is still possible that other mutations such as R140W might influence disease (primary open angle glaucoma) by mechanisms distinct from R345W or L451F, and therefore may behave differently. Another interesting finding in our study was the Q226H F3 variant. Although this variant is predicted to change *EFEMP1* splicing (ClinVar), it was surprising to observe a ~ 30 kDa truncated product, especially since our expression constructs lack introns (Sup. Figure 1). Additional studies will be needed to uncover the origin of this product as well as investigate the mechanisms at play that ultimately lead to inherently lower transcript, intracellular, and secreted Q226H levels.

One limitation of our study is that it primarily utilized HEK293A cells. While these cells likely share most of the major protein synthesis, folding and degradation machinery as retinal cells that express F3 (i.e., RPE cells), and are an appropriate model for initial F3 variant screening, we acknowledge that more physiologically relevant model systems are currently accessible. Accordingly, follow-up studies will be focused on evaluating intriguing variants (e.g., L451F) in more relevant model systems, such as CRISPR-modified induced pluripotent stem cells (iPSCs) that have been differentiated into RPE cells or F3 variants knocked into mice to develop an in vivo model system for further analysis.

Overall, little is known about the molecular and cellular influences that regulate F3 secretion and function. Mapping and testing F3 mutations as we have performed, combined with comprehensive ocular phenotypic data, will provide more information regarding whether there are certain ‘hot-spots’ in F3 that are prone to secretion-compromising or pathogenic mutations. For example, the combination of analogous studies^40–43^ have led to the determination that the olfactomedin (OLF) domain is a ‘hot-spot’ for many pathogenic mutations in myocilin (MYOC). Likewise, we noticed a trend that C-terminal mutations (i.e., mutations occuring after N249, as an approximate midpoint) were more prone to either secretion defects (e.g., R345W and L451F), or further destabilization (i.e., N249Q/Y397H). This approximate region of F3 (amino acids 259–493) has also been shown to bind to tissue inhibitor of matrix metalloproteinase 3 (TIMP3)^44^, a critical extracellular matrix regulatory component that is also associated with Sorsby’s Fundus Dystrophy^45^. Failure of F3 to bind to TIMP3 or other known F3 interacting partners^46,47^ due to partial misfolding in its C-terminus may ultimately influence its fate in the cell and at the organismal level. Additionally, recent studies have indicated that the C-terminus of F3 may be the source of amyloid fibrils found in the veins of aged individuals^48^, suggesting that this portion of the protein has a propensity for β-sheet formation and aggregation.

While little in-depth knowledge exists for patients with the L451F mutation, we do know that this mutation was identified in two separate unrelated heterozygous individuals, one with retinal dystrophy and the other with congenital nystagmus and otherwise poor ocular workup (information provided by Eurofins Clinical Diagnostics, personal communication). Given our observations of L451F F3 secretion and destabilization, combined with the identification in multiple individuals with eye disease, it is intriguing to speculate that L451F might be a disease modifier or actually trigger ocular disease.

The C-terminal F3 fragility hypothesis doesn’t preclude the idea that N-terminal mutations couldn’t be detrimental to F3 folding and function. In fact, we would predict that the recently identified C55R F3 mutation, located in the atypical calcium-binding EGF domain of F3 and associated with Marfanoid syndrome^49^, could substantially effect F3 secretion and/or redox/disulfide state. Yet, it is important to mention that it is still unclear how the other F3 mutations such as R345W cause diseases such as ML—whether it is due to the escape of misfolded F3 from the cell which wreaks havoc in the extracellular matrix, possibly triggering complement activation^50^, or whether it is due to accumulation of poorly folded intracellular F3, or a combination of both of these possibilities. Ultimately, in the absence of multiple sets of reliable phenotypic patient data, future studies aimed at testing the effects of newly identified (e.g., L451F, Y397H) and rationally-designed (e.g., C338A) mutants in mice will be critical for developing a better understanding of the role of misfolded F3 and its relation to ocular diseases.

## Methods

### In silico screening of variants

F3 (*EFEMP1*) variants were identified using the ClinVar website (https://www.ncbi.nlm.nih.gov/clinvar/). The protein-level consequences of these alterations were then assessed by PROVEAN (http://provean.jcvi.org/index.php) and PolyPhen 2.0 (http://genetics.bwh.harvard.edu/pph2/). Allele frequency of the variation was determined by gnomAD (https://gnomad.broadinstitute.org/, V2.1.1, Ensembl gene ID: ENSG00000115380.14, region: 2.56093102–56151274). Clustal Omega (https://www.ebi.ac.uk/Tools/msa/clustalo/) was used to align Uniprot sequences (https://www.uniprot.org/).

### Plasmid generation

N-terminal FLAG-tagged (FT) F3 constructs were either generated from pcDNA FT WT F3 or pENTR1A FT WT F3 templates using the Q5 mutagenesis kit (New England Biolabs, Ipswich, MA, USA). pENTR1A constructs were then shuttled into the pcDNA DEST40 vector (Life Technologies, Carlsbad, CA, USA) or pLenti CMV Puro DEST (gift from Eric Campeau and Paul Kaufman, Addgene plasmid # 17452) by an LR clonase II reaction (Life Technologies) to generate the final construct. The pcDNA FT WT F3 construct is shown in Sup. Figure 1, and uses a preprotrypsin leader sequence followed by the FLAG peptide, and then the F3 sequence. The plasmid map was made using SnapGene 5.1.7 (GSL Biotech, San Diego, CA, USA). All F3 mutations and plasmids were verified by Sanger sequencing.

### Cell culture and transfection

Human embryonic kidney cells (HEK293A, Life Technologies) were cultured at 37 °C with 5% CO_2_ in Dulbecco’s minimal essential medium (DMEM) supplemented with high glucose, (4.5 g/L, Corning, Corning, NY, USA), 10% fetal bovine serum (FBS, Omega Scientific, Tarzana, CA, USA) and 1% penicillin–streptomycin-glutamine (Gibco, Waltham, MA, USA). Cells were plated at a density of 100,000 cells/well in a 24 well plate and transfected the following day with 500 ng of midi-prepped endotoxin-free plasmid DNA (Qiagen, Germantown, MD, USA) using Lipofectamine 3000 (Life Technologies) as described previously^51^. Forty-eight hours after transfection, fresh serum-free media was added. Cells were harvested and media was collected 24 h later (72 h post transfection). Human immortalized retinal pigmented epithelial cells (ARPE-19, CRL-2302, American Type Culture Collection, Manassas, VA, USA) were cultured in DMEM/F12 media supplemented with 10% fetal bovine serum (FBS, Omega Scientific, Tarzana, CA, USA), HEPES (Corning) and penicillin/streptomycin and glutamine (PSQ, Gibco, Germantown, MD, USA). To generate stably expressing F3 cell lines, ARPE-19 cells were infected with VSV-G-pseudotyped lentivirus packaged with the pLenti CMV Puro vector containing FT F3 variants. Stable populations were selected using puromycin. Cells were plated at a density of 150,000 cells/well in a 12 well plate, changed to serum free media after 24 h, and harvested 24 h later (48 h post plating).

### Western blot

Cells were washed with Hanks buffered salt solution (HBSS, Sigma-Aldrich, St. Louis, MO, USA), then lysed with radioimmunoprecipitation assay (RIPA) buffer (Santa Cruz, Dallas, TX, USA) supplemented with Halt protease inhibitor (Pierce, Rockford, IL, USA) and benzonase (Millipore Sigma, St. Louis, MO, USA) for 3–5 min at room temperature, and then spun at max speed (14,800 rpm) at 4 °C for 10 min. The soluble supernatant was collected and protein concentration was quantified via bicinchoninic assay (BCA) (Pierce). Twenty to thirty µg of soluble supernatant or 20 μL of conditioned media was run on a 4–20% Tris-Gly SDS-PAGE gel (Life Technologies) and transferred onto a nitrocellulose membrane using an iBlot2 device (Life Technologies). After probing for total transferred protein using Ponceau S (Sigma-Aldrich), membranes were blocked overnight in Odyssey Blocking Buffer (LICOR, Lincoln, NE, USA). Membranes were probed with rabbit anti-FLAG (1:5000; Thermo Fisher Scientific, Waltham, MA, USA, cat# PA1-984B) or mouse anti-β-actin (1:1000; Sigma-Aldrich, cat# A1978). All Western blot imaging was performed on an Odyssey CLx (LI-COR) and band quantification was performed using Image Studio software (LI-COR).

### Quantitative PCR

Transfected HEK293A or stably expressed F3 ARPE-19 cells were trypsinized (0.25% Trypsin EDTA, Gibco), quenched with full DMEM or DMEM/F12 media, respectively, and centrifuged at max speed (3,000 rpm) at 4 °C for 10 min. Cell pellets were washed with HBSS, centrifuged again, then RNA extraction from cell pellets was performed using the Aurum Total RNA Mini Kit (BioRad). 315–400 ng RNA (HEK293A) or 10–50 ng RNA (ARPE-19) was reverse transcribed using qScript cDNA SuperMix (Quanta Bioscience, Beverly, MA, USA) and the cDNA was diluted 5X in DNase/RNase-free water. cDNA was amplified with TaqMan Fast Advanced Master Mix (Thermo Fisher) and transcripts were detected using h*HSPA5* (cat# hs00607129_gH), h*DNAJB9* (cat# hs01052402_m1), h*ASNS* (cat# hs04186194_m1), and h*ACTB* (cat# hs01060665_g1) TaqMan probe sets. For quantifying F3 (*EFEMP1*) mRNA levels, cDNA was amplified with PowerUp SYBR Green Master Mix (Thermo Fisher). Transcripts were amplified using h*EFEMP1* forward (5′ GGGGATCCTTTGCATGTCAG) and reverse (5′ TGAAACCCAGGACTGCACTG) primers, using *RPLP2* forward (5′ CGTCGCCTCCTACCTGCT) and reverse (5′ CCATTCAGCTCACTGATAACCTTG) primers as a housekeeping gene. Amplification for both TaqMan assays and Sybr Green was performed on a QuantStudio 6 and visualized and quantified using the associated software (Thermo Fisher).

### Statistical analysis

To determine statistical significance, samples were compared using a one-sample *t* test using Excel against a hypothetical value of 1 (i.e., unchanged compared to the control). Significance was set at *p < 0.05, **p < 0.01, and ***p < 0.001.

## Supplementary Information

Supplementary Information
