# Supplementary material for: Clinically-identified C-terminal mutations in fibulin-3 are prone to misfolding and destabilization
Source: Sci Rep. 2021 Feb 4;11:2998. doi: 10.1038/s41598-020-79570-x (PMC7862258; doi:10.1038/s41598-020-79570-x)

CLINICALLY-IDENTIFIED C-TERMINAL MUTATIONS IN FIBULIN-3 ARE PRONE TO MISFOLDING AND DESTABILIZATION

DaNae R. Woodard[1], Emi Nakahara[1] and John D. Hulleman[1, 2, Ω]

[1] Department of Ophthalmology, University of Texas Southwestern Medical Center, 5323 Harry Hines Blvd, Dallas, Texas, United States

[2] Department of Pharmacology, University of Texas Southwestern Medical Center, 5323 Harry Hines Blvd, Dallas, Texas, United States

[Ω]Tel: 1 214 648 3677; Fax: 1 214 648 9061

[Ω]Email: John.Hulleman@UTSouthwestern.edu

SUPPLEMENTAL FIGURE LEGENDS

Supplemental Figure 1. pcDNA FT F3 vector for F3 variants. Plasmid map was generated using SnapGene version 5.1.7, https://www.snapgene.com/.

Supplemental Figure 2. F3 (*EFEMP1*) transcript levels of mutants in (A) HEK293A and (B) ARPE-19 cells. (*A*) qPCR analysis of h*EFEMP1* transcript levels with SYBR Green *EFEMP1* primers in WT F3, R345W, C338A, and L451F-expressing cells. (B) qPCR analysis of h*EFEMP1* transcript levels with SYBR Green *EFEMP1* primers in WT F3, D49A, R140W, R345W, Y397H, and L451F-expressing cells. n = 3, mean ± SEM (one sample t-test vs. hypothetical value of 1 [i.e., unchanged]). n = 5, mean ± SEM (* - $p<0.05$, ** - $p<0.01$, one sample t-test vs. hypothetical value of 1 [i.e., unchanged]).

Supplemental Figure 3. Secretion propensities of F3 in ARPE-19 cells. (*A*) Western blot of secreted and intracellular levels of F3 variants stably expressed in ARPE-19 cells. (*B*) Secretion and intracellular levels of F3 variants in ARPE-19 cells and (C) their corresponding secretion propensities, n = 6, mean ± SEM (* - $p<0.05$, ** - $p<0.01$, *** - $p<0.001$, one sample t-test vs. hypothetical value of 1 [i.e., unchanged]).

Supplemental Figure 4. Protein alignment near the L451 residue of F3. (A) Clustal Omega alignment of F3 proteins across the indicated species. (B) Clustal Omega alignment of different fibulin proteins at the L451 position.

Supplemental Figures 5 and 6. Full western blots from Fig. 2, 4, 5 and Supplemental Fig. 3.

Supplemental Fig. 1

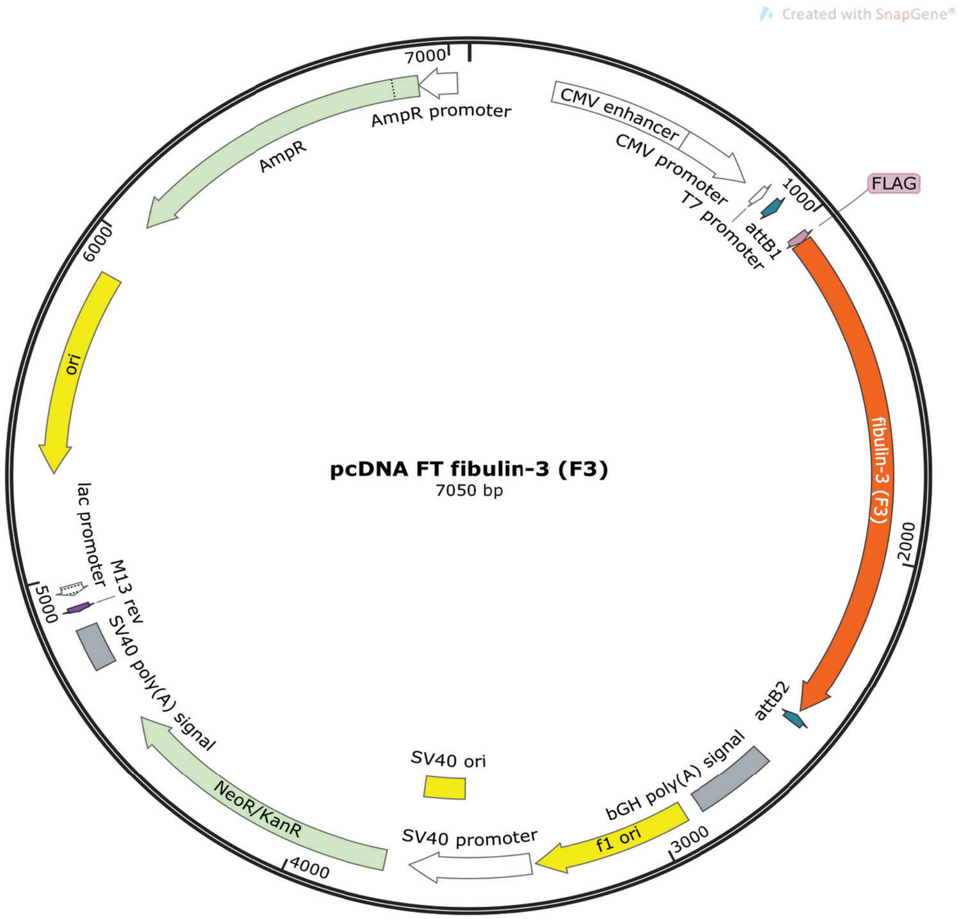

Supplemental Fig. 2

A

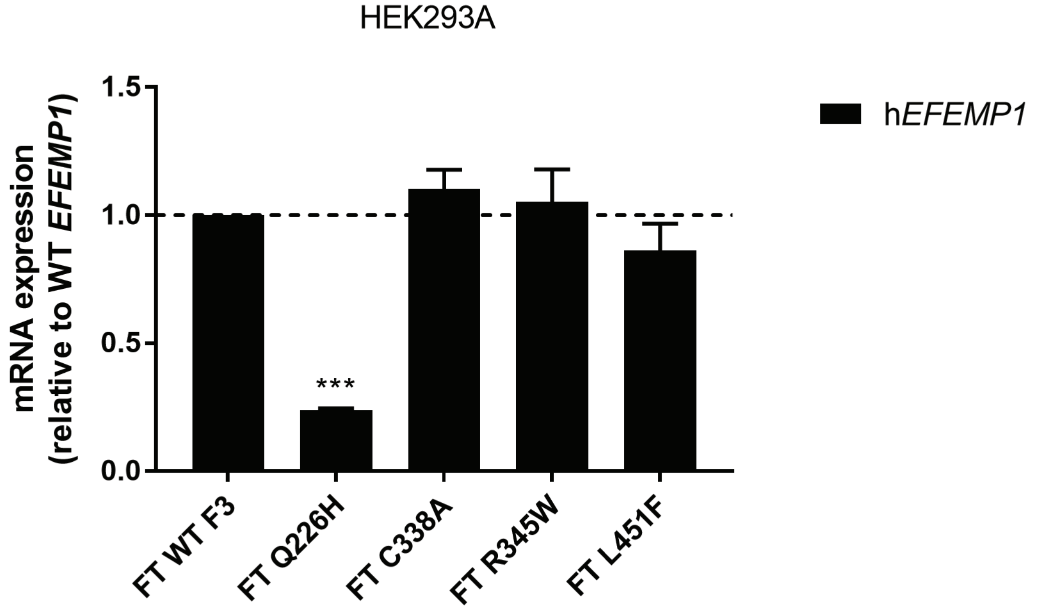

B

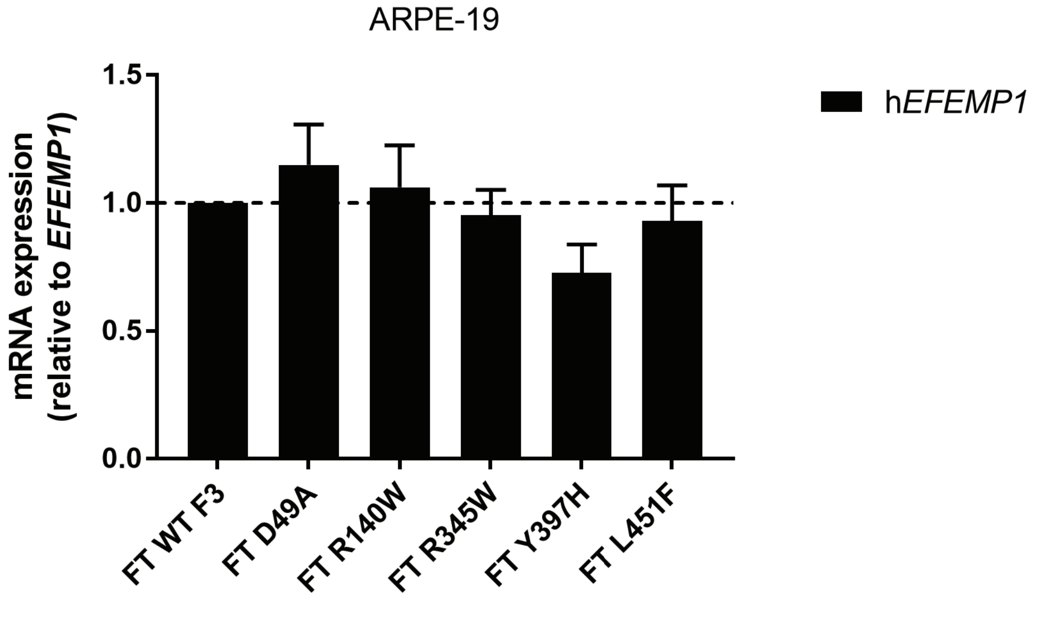

Supplemental Fig. 3

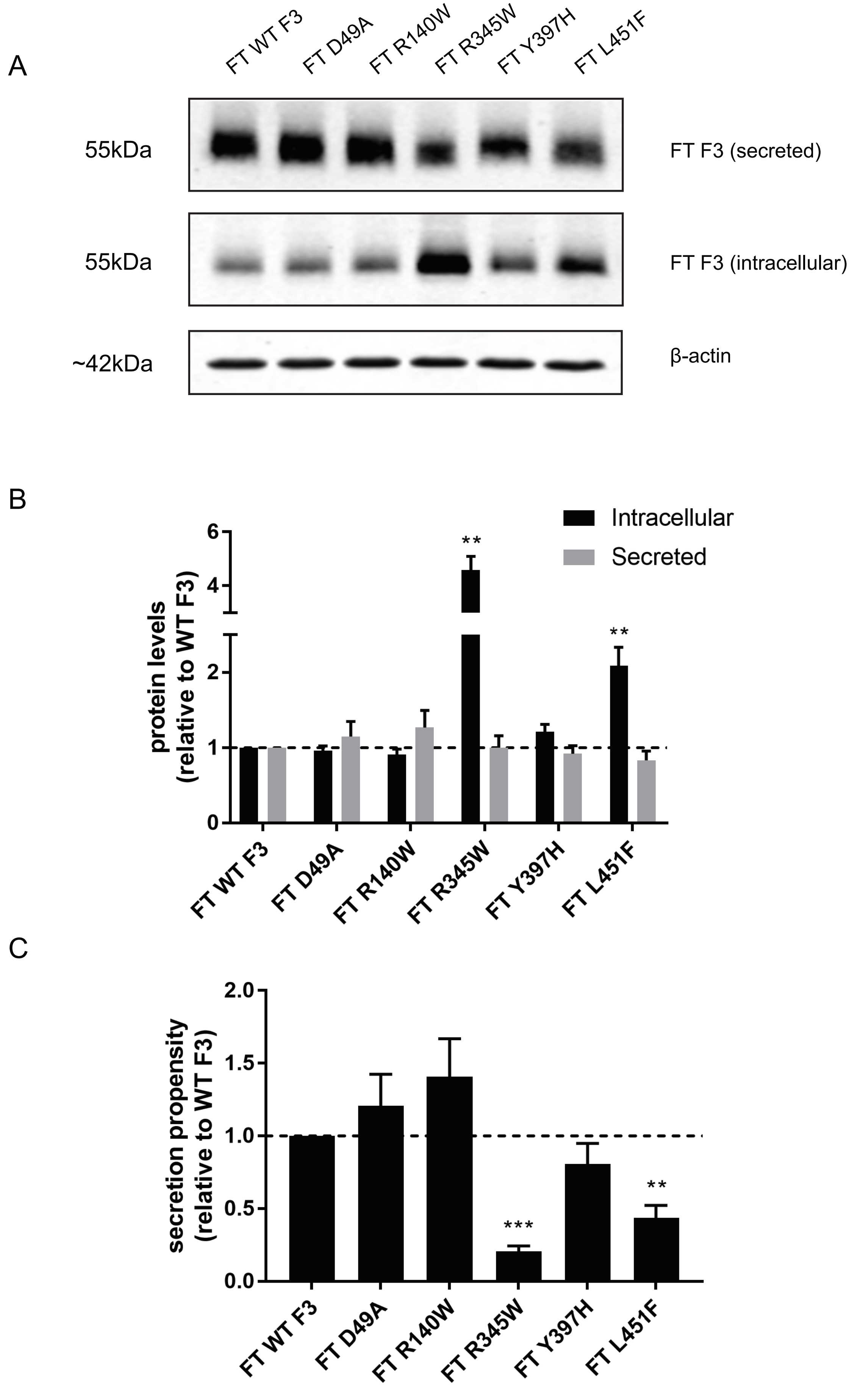

Supplemental Fig. 4

A

```
                                                        L451

human           TTIYANTINTFRIKSGNENGEFYLRQTSPVSAMLVLVKSLSGPREHIVDLEMLTVSSIGT  475
rat             TTIYANTINTFRIKSGNENGEFYLRQTSPVSAMLVLVKSLTGPREHIVGLEMLTVSSIGT  475
mouse           TMIYANTINTFRIKSGNENGEFYLRQTSPVSAMLVLVKSLSGPREYIVDLEMLTVSSIGT  475
horse           TTIYANTINTFRIKSGNENGEFYLRQTSPVSAMLVLVKSLSGPREYIVDLEMLTVNSLGT  596
rabbit          TTIYANTINTFRIKSGNENGEFYLRQTSPVSAMLVLVKSLTGPREYIVDLEMLTVNSIGT  476
macaque         TTIYANTINTFRIKSGNENGEFYLRQTSPVSAMLVLVKSLSGPREHIVDLEMLTVSSIGT  475
turtle          TTIYPNTINTFRIKSGNENGEFYLRQTSAISAMLVLVKSLSGPREHIVDLEMLTVNSLN-  431
bird            TTIYPNTINTFRIKSGNENGEFYLRQTSAVSAMLVLVKSLSGPREHIVDLEMLTVNSLN-  432
chicken         TTIYPNTINTFRIKSGNENGEFYLRQTSAVSAMLVLVKSLSGPREHIVDLEMLTVNSLN-  433
                * ** *********************** :**********:****:**.******.*:.
```

B

```
                        L451

fibulin-1       GMTVGVVRQVRPIVGPFHAVLKLEMNYVVG-GVVSHRNVVNVHIFVSEYWF  703
fibulin-2       NAYTGVVYLQRAVLEPRDFALDVEMKLWRQ-GSVTT-FLAKMHIFFTTFAL  1184
fibulin-3       SPVSAMLVLVKSLSGPREHIVDLEMLTVSSIGTFRTSSVLRLTIIVGPFSF  493
fibulin-4       NNVSAMLVLARPVTGPREYVLDLEMVTMNSLMSYRASSVLRLTVFVGAYTF  443
fibulin-5       GPISATLVMTRPIKGPREIQLDLEMITVNTVINFRGSSVIRLRIYVSQYPF  448
                .   . :   : :  * .  :.:**             : .: : .  : :
```

Supplemental Fig. 5

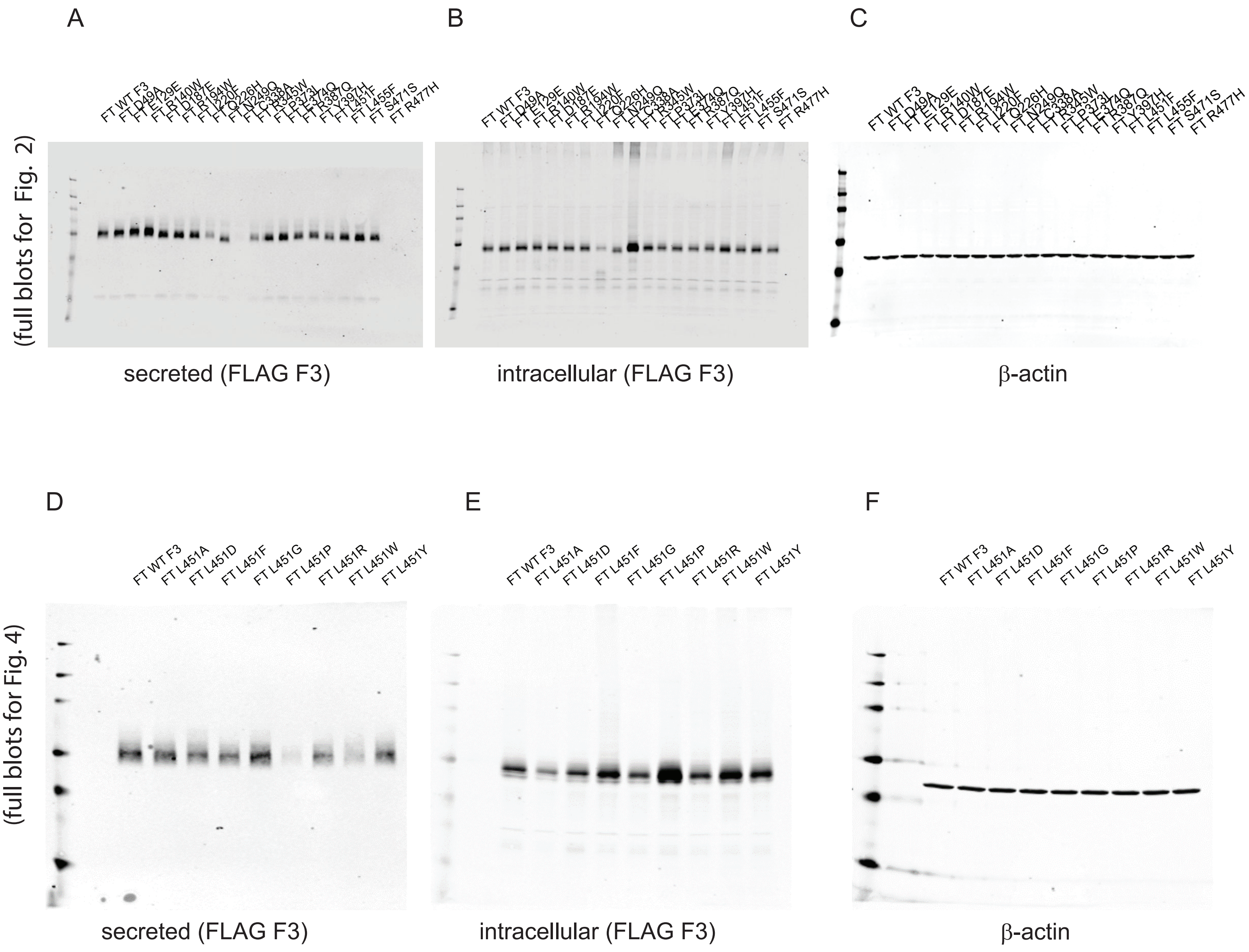

Supplemental Fig. 6

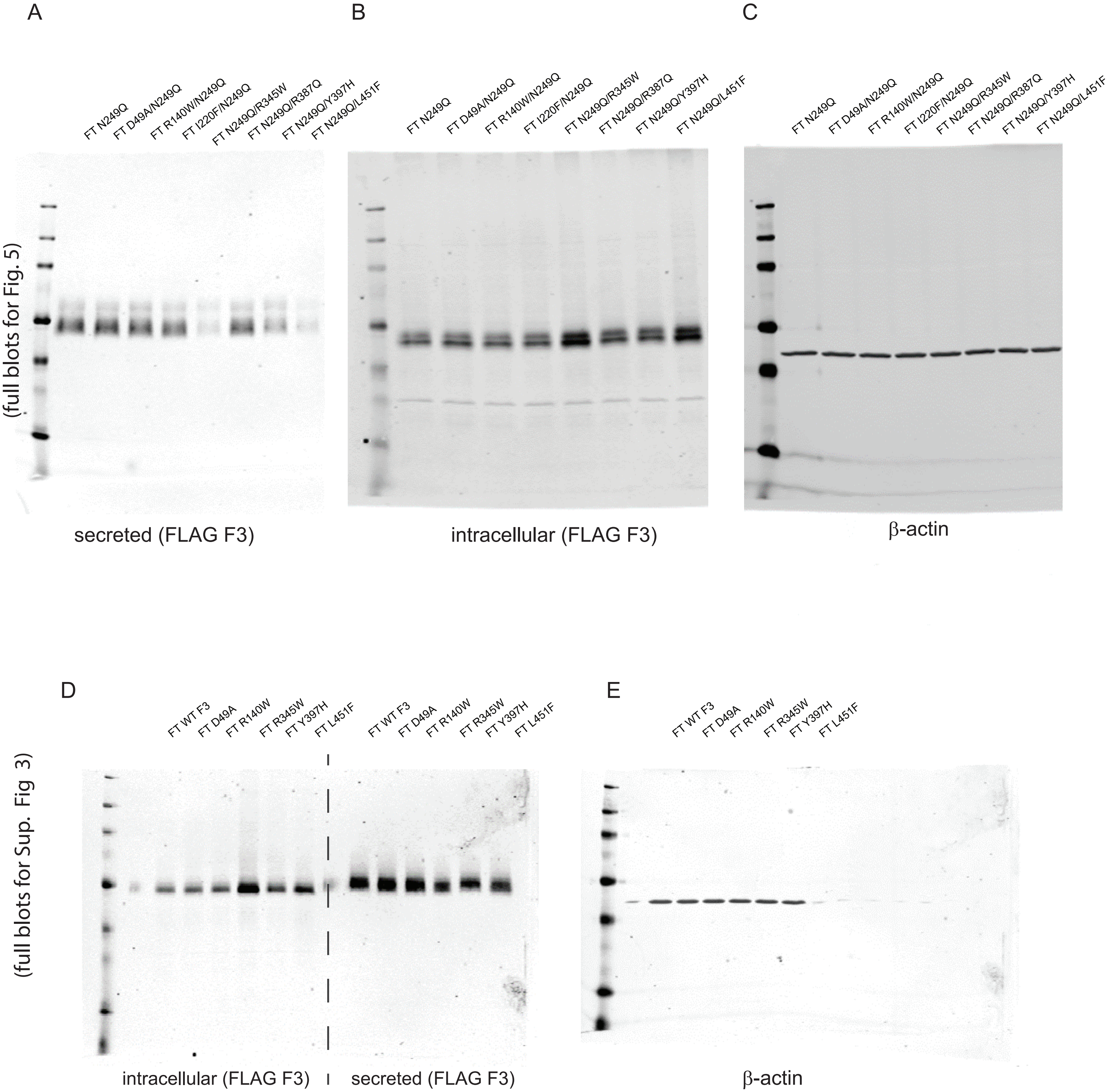

Supplement: Supplementary file 1 — Supplementary Information [file 41598_2020_79570_MOESM1_ESM.pdf]
